# ARHGAP10 inhibits the epithelial–mesenchymal transition of non-small cell lung cancer by inactivating PI3K/Akt/GSK3β signaling pathway

**DOI:** 10.1186/s12935-021-02022-7

**Published:** 2021-06-26

**Authors:** Lan-Lan Lin, Fan Yang, Dong-Huan Zhang, Cong Hu, Sheng Yang, Xiang-Qi Chen

**Affiliations:** 1 Department of Respiratory Medicine, Fujian Medical University Union Hospital, Fuzhou, 350001 Fujian People’s Republic of China; 2 Department of Oncology, Fujian Medical University Union Hospital, Fuzhou, 350001 Fujian People’s Republic of China

**Keywords:** ARHGAP10, Non-small cell lung cancer, Epithelial–mesenchymal transition, PI3K/Akt/GSK3β

## Abstract

**Background:**

Rho GTPase activating protein 10 (ARHGAP10) has been implicated as an essential element in multiple cellular process, including cell migration, adhesion and actin cytoskeleton dynamic reorganization. However, the correlation of ARHGAP10 expression with epithelial–mesenchymal transition (EMT) in lung cancer cells is unclear and remains to be elucidated. Herein, we investigated the relationship between the trait of ARHGAP10 and non-small cell lung cancer (NSCLC) pathological process.

**Methods:**

Immunohistochemistry was conducted to evaluate the expression of ARHGAP10 in NSCLC tissues. CCK-8 assays, Transwell assays, scratch assays were applied to assess cell proliferation, invasion and migration. The expression levels of EMT biomarkers and active molecules involved in PI3K/Akt/GSK3β signaling pathway were examined through immunofluorescence and Western blot.

**Results:**

ARHGAP10 was detected to be lower expression in NSCLC tissues compared with normal tissues from individuals. Moreover, overexpression of ARHGAP10 inhibited migratory and invasive potentials of A549 and NCI-H1299 cells. In addition, ARHGAP10 directly mediated the process of EMT via PI3K/Akt/GSK3β pathway. Meanwhile, activation of the signaling pathway of insulin-like growth factors-1 (IGF-1) reversed ARHGAP10 overexpression regulated EMT in NSCLC cells.

**Conclusion:**

ARHGAP10 inhibits the epithelial–mesenchymal transition in NSCLC via PI3K/Akt/GSK3β signaling pathway, suggesting agonist of ARHGAP10 may be an optional remedy for NSCLC patients than traditional opioids.

## Background

Lung cancer remains the most prevalent cause of cancer-related mortality worldwide [1]. The predominant pathological subtype is non-small cell lung cancer (NSCLC), accounting for nearly 85% of malignant pulmonary tumors [2]. Given its highly invasive and metastatic property, the five-year survival rate is only 4–17% depending on stage and region difference [3, 4]. Despite the recent essential elucidation on key driver genes and core signaling pathways [5], much remains to be uncovered to better comprehension of NSCLC molecular mechanism. Therefore, identification of the potential pathogenic genes is urgently required for therapeutic progress of lung cancer.

Rho GTPase activating protein 10 (ARHGAP10; also known as ARHGAP21), a member of Rho GTPase activating protein (RhoGAP) family, possesses GAP domain, pleckstrin homology (PH) domain and PDZ domain [6]. The existence of GAP domain presents the major GTPase inhibition activity in modulating cell migration [7]. ARHGAP10 has been implicated as an essential element in multiple cellular process, including cell migration, adhesion and actin cytoskeleton dynamic reorganization [8]. Emerging evidence showed the critical integration of ARHGAP10 in epithelial–mesenchymal transition (EMT) and metastasis in cancer progression [9, 10]. Epithelial–mesenchymal transition, defined as the loss of epithelial characteristics and the acquisition of mesenchymal phenotype, confers tumor cells with abilities essential for drug resistance, metastasis and acquired stem cell traits [11, 12]. It has been demonstrated that EMT involvement in morphogenesis and metastasis of cancers, especially in lung cancer [13]. However, the correlation of ARHGAP10 expression with EMT of lung cancer cells is unclear and remains to be elucidated. Herein, we investigated the relationship between the trait of ARHGAP10 and NSCLC pathological process.

In the present study, our results elaborated the expression of ARHGAP10 was significantly decreased in NSCLC cells and tissues. Moreover, we demonstrated that ARHGAP10 suppressed EMT by inactivation of PI3K/Akt pathway in NSCLC cells. Our findings suggested agonist of ARHGAP10 may be an optional remedy for NSCLC patients than traditional opioids.

## Materials and methods

### Patients and tissue samples

All clinical samples including 66 malignant and 66 non-neoplastic lung tissues were obtained from the tissue bank of Fujian Medical University Union Hospital. The application of archived cancer samples was approved by the relevant Ethics Commission. In the present study, there was no patient received radiotherapy or chemotherapy prior to surgery. All excised specimens were stored − 80 °C for long‑term conservation.

### Immunohistochemistry (IHC)

Immunohistochemistry was conducted on formalin-fixed paraffin-embedded specimens. Antigen retrieval was conducted in 10 mM citrate buffer (pH = 6.0) for 10 min after deparaffinization. Anti-ARHGAP10 primary antibody (Santa Cruz, CA, USA) was incubated at 4 °C overnight and HRP-labeled broad-spectrum secondary antibody for 30 min at room temperature. After peroxidase substrate DAB staining, slices were counterstained with hematoxylin for 3 min and final images were captured by inverted microscope (OLYMPUS CKX41, Japan). The degree of immunostaining was reviewed and scored based on the intensity of staining: 0 (no staining), 1 (weak staining = light brown), 2 (moderate staining = brown) and 3 (strong staining = dark brown). Moderate and strong staining were considered as ARHGAP10 positive, while no and weak staining were considered as negative.

### Cell culture

Lung epithelial cell line BEAS-2B and NSCLC cell lines containing A549, NCI-H1299, H1975, SKMES-1 were purchased from Cell Bank of Chinese Academy of Science (Shanghai, China). All cells were cultured in RPMI-1640 medium (HyClone, South-Logan, UT, USA) containing 10% fetal bovine serum (FBS) (Gibco, Grand Island, NY, USA) and 1% penicillin–streptomycin (Beyotime, Tianjin, China) at a 37 °C humidified incubator with 5% CO_2_ (Thermo Scientific, Waltham, MA, USA). The medium was replaced during incubation based on the cellular demand.

### Lentivirus infection

Lentiviral construction of ARHGAP10 overexpression (OE) vectors or corresponding empty vector were generated by Genechem Co., LTD. (Shanghai, China). A549 cells and NCI-H1299 cells were seeded into six-well plates at density of 3.0 × 10^5^ cells/well overnight. When the confluence reached about 20%, cells were infected by ARHGAP10 lentivirus or control lentivirus in the presence of 8 μg/ml polybrene with 10 multiple of infection (MOI). After infection for 16 h, the medium containing virus particles was removed and changed to complete medium. Three days post-transfection, GFP expression was observed in three randomly-selected fields using a fluorescence microscope (magnification, ×200). About 90% incubated cells observed GFP staining was considered to be feasible for the following procedure. Optimal concentration of puromycin (Sigma, St. Louis, MO, USA) was confirmed in preliminary experiment and the final concentration was determined as 4.0 μg/ml. We further added 4.0 μg/ml puromycin into six-well plates for 48 h to select infected cells. To preserve the property of stable transgenic plants, the presence of 2.0 μg/ml puromycin were used for following assays. Mixed clone pool was collected and ARHGAP10 expression were determined by RT-qPCR and Western blot.

### Reverse transcription‑quantitative PCR (RT‑qPCR)

RNAs were extracted from cell lines using TRIzol® reagent (Invitrogen, Thermo Fisher Scientific, Inc.) according to the manufacturer’s protocol. After quantification and RNA purity were confirmed, the extracted RNA was reverse transcribed into cDNA using Reverse Transcription Kit (Fermentas, Vilnius, Lithuania). Then, RT-qPCR was conducted on ABI 7300 Real-Time PCR system (Applied Biosystems, Foster City, CA, USA) with a SYBR Green PCR Kit (Thermo Fisher Scientific, Inc.) according to the following primer sequences: ARHGAP10 forward, 5ʹ-ACTGAAACCCTGATTAAACC-3ʹ and reverse, 5ʹ-ATCTGCCTCTTGTAAATGTG-3ʹ; GAPDH forward, 5ʹ-CACCCACTCCTCCACCTTTG-3ʹ and reverse, 5ʹ-CCACCACCCTGTTGCTGTAG-3ʹ. The relative gene expression level was measured using the 2^−ΔΔCt^ method.

### Western blot

Total proteins were isolated using RIPA buffer (Solarbio, Beijing, China) containing protease and phosphatase inhibitors. The protein concentration was quantified by BCA Protein Assay Kit (Thermo Fisher Scientific, Waltham, MA, USA). Approximately 20 µg of total proteins were separated by SDS-PAGE and transferred into PVDF membranes (Millipore, Billerica, MA, USA). After blockage with 10% non-fat milk, the membranes were probed with primary antibodies overnight at 4 °C with gentle shaking. Next day, after washing, corresponding secondary antibodies were incubated at room temperature for 1 h. Later, membranes were washed again and detected using standard chemiluminescence (Bio-Rad, Richmond, CA, USA) and the Bio-Rad ChemiDoc™XRS System.

### Antibodies

The following antibodies were applied: Anti-ARHGAP10 (Catalog No. sc-390145, Santa Cruz). Anti-E-Cadherin (Catalog No. ab40772, abcam). Anti-N-Cadherin (Catalog No. ab76011, abcam). Anti-Vimentin (Catalog No. ab92547, abcam). Anti-Snail (Catalog No. ab216347, abcam). Anti-PI3K (Catalog No. 4249, Cell Signaling Technology). Anti-Akt (Catalog No. 4691, Cell Signaling Technology). Anti-phospho-Akt (Catalog No. 4060, Cell Signaling Technology). Anti-GSK3β (Catalog No. ab32391, abcam). Anti-phospho-GSK3β (Catalog No.9323, Cell Signaling Technology). Anti-β-catenin (Catalog No. 8480, Cell Signaling Technology). Anti-GAPDH (Catalog No. ab181602, abcam).

### Cell proliferation assay

Cell Counting Kit-8 (CCK-8) (Beyotime, Tianjin, China) was applied to assess cell proliferation. Cells were seeded in 96-well plates at a density of 3 × 10^3^ cells/well and cultured overnight. After incubation for 0, 24, 48 and 72 h, 100 µl mixture of CCK-8 and serum-free medium at a volume ratio of 1:10 was added and incubated for additional 1 h at 37 °C. The absorbance value (OD) was measured at 450 nm.

### Transwell invasion and migration assay

Cellular invasive ability was determined by Transwell assay. Upper Transwell chambers were either uncoated for the migration assay or precoated with 30 µg Matrigel (Corning, New York, NY, USA) for the invasion assay and clotted in a 37 °C incubator for 30 min. The density of cells was adjusted to 5.0 × 10^4^ cells/well for the migration assay, 1.0 × 10^5^ cells/well for the invasion assay and resuspended in serum-free medium, RPMI-1640 medium containing 10% FBS was applied to the lower chambers. After incubation at 37 °C for 24 h, non-invaded cells on the upper chambers were scraped out. Cells on the lower chambers were fixed in 4% paraformaldehyde and stained with 1% crystal violet (Solarbio Life Sciences, Beijing, China) for 15 min. The number of cells were counted under microscope from at least 3 random fields at a magnification of 200×.

### Scratch assay

Cells were plated into the 6-well plate with complete medium. When cells reached full confluence, scratch wounds were scraped in a straight line using a 200 μl pipette tip. Cell debris was then removed using phosphate buffered saline (PBS) and serum-free medium was replaced. Photographs were captured under Leica DMi8 inverted microscope at 0 h and 24 h respectively. The scratch healing rate was calculated as follows: (scratch area of 0 h − scratch area of 24 h)/scratch area of 0 h × 100%.

### Immunofluorescence (IF)

A549 and NCI-H1299 cells were grown on the coverslips and fixed with 4% fresh paraformaldehyde for 20 min at room temperature. After permeabilization with 0.5% Triton X-100 for 20 min and blockage with 5% bovine serum albumin (BSA) for 1 h, cells were incubated with 50 µl ARHGAP10, E-cadherin or vimentin antibody diluted in PBS (1:50) (Santa Cruz, CA, USA) overnight at 4 °C. Then, cells were stained with FITC-conjugated or PE-conjugated secondary antibody for 1 h at room temperature in dark. After washing, cells were counterstained with DAPI for 10 min and mounted with ProLong Gold Anti-Fade Mounting Medium with DAPI (Molecular Probes). Images were generated using confocal laser-scanning microscope (LSM 510, Carl Zeiss, Welwyn Garden City, UK).

### Statistical analysis

All above experiments were conducted in triplicate independently and data were presented as the mean ± standard deviation (SD). Statistical analyses were performed using GraphPad Prism 7.0 software (La Jolla, CA, USA), Photoshop CS4 (San Jose, CA, USA) and Image J software (NIH). Differences between groups were evaluated by Student’s t-test or one-way ANOVA analysis. *p* value of < 0.05 was considered statistically significant.

## Results

### Expression and clinical significance of ARHGAP10 in NSCLC patients

**Fig. 1:**
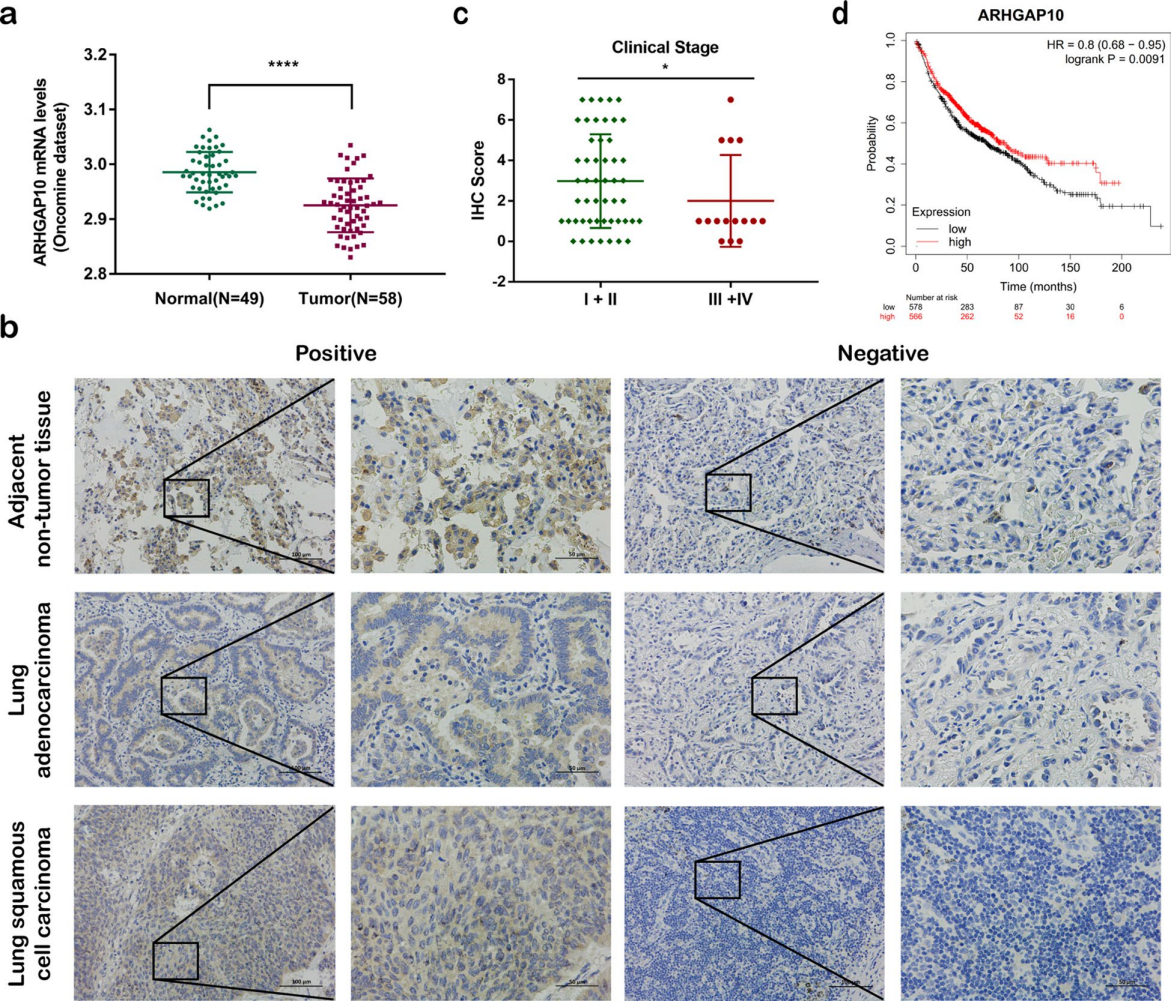
ARHGAP10 expression in NSCLC tissues. **a** ARHGAP10 was downregulated in NSCLC tissues compared with the normal tissues in Oncomine dataset. **b** Relative protein expression of ARHGAP10 in NSCLC tissues (n = 66) compared with adjacent non-tumor tissues (n = 66) with immunohistochemistry. **c** IHC scores of ARHGAP10 staining in NSCLC clinical stage. **d** Kaplan–Meier survival analysis about ARHGAP10 expression on 1144 NSCLC patients originated from TCGA. (*p < 0.05; ****p < 0.0001)

To examine the expression of ARHGAP10 in NSCLC, we analyzed RNA-Seq data from Oncomine Database (www.oncomine.org). Strikingly, ARHGAP10 was found to be lower expression in NSCLC tissues compared with normal tissues from individuals (*p* < 0.0001) (Fig. 1a). Then we further detected ARHGAP10 protein expression in 66 NSCLC tissues and 66 adjacent non-tumorous epithelial tissues with immunohistochemistry. Representative IHC images for ARHGAP10 expression in NSCLC and adjacent non-tumor tissues were shown in Fig. 1b. According to the relevant ARHGAP10 expression in tumor tissues, 66 NSCLC patients were classified into two groups: negative group (n = 30) and positive group (n = 36), the correlation between ARHGAP10 expression and clinicopathological characteristics in NSCLC patients were shown in Table 1. Expression of ARHGAP10 was identified to be correlated with lymphnode metastasis (*p* = 0.043) and TNM stage (*p* = 0.019) but not with other clinicopathological characteristics in patients with NSCLC. The corresponding IHC scores of ARHGAP10 staining in NSCLC clinical stage was presented in Fig. 1c. We further analyzed the relationship between ARHGAP10 expression and prognosis in NSCLC patients using Kaplan–Meier survival curves from The Cancer Genome Atlas (TCGA, http://www.cancer.gov/) database. The 1144 NSCLC patients were classified into two groups: ARHGAP10 high expression group (n = 566) and ARHGAP10 low expression group (n = 578). The Kaplan–Meier analysis indicated that high expression of ARHGAP10 correlated with a favorable prognosis (HR = 0.8, Logrank *p* = 0.0091, Fig. 1d).

**Table 1:** Correlation of ARHGAP10 expression with NSCLC clinicopathological characteristics

Characteristics	No. of patients	ARHGAP10 expression		*p* value
		Negative	Positive	
Age				
≥ 60	34	14	20	0.621
< 60	32	16	16	
Gender				
Male	39	15	24	0.213
Female	27	15	12	
Histological type				
LUAD	50	26	24	0.084
LUSC	16	4	12	
Lymphnode metastasis				
Absent	42	15	27	0.043*
Present	24	15	9	
TNM stage				
I + II	51	19	32	0.019*
III + IV	15	11	4	

*LUAD* Lung adenocarcinoma, *LUSC* Lung squamous cell carcinoma (*p < 0.05)

### ARHGAP10 overexpression inhibits NSCLC progression

**Fig. 2:**
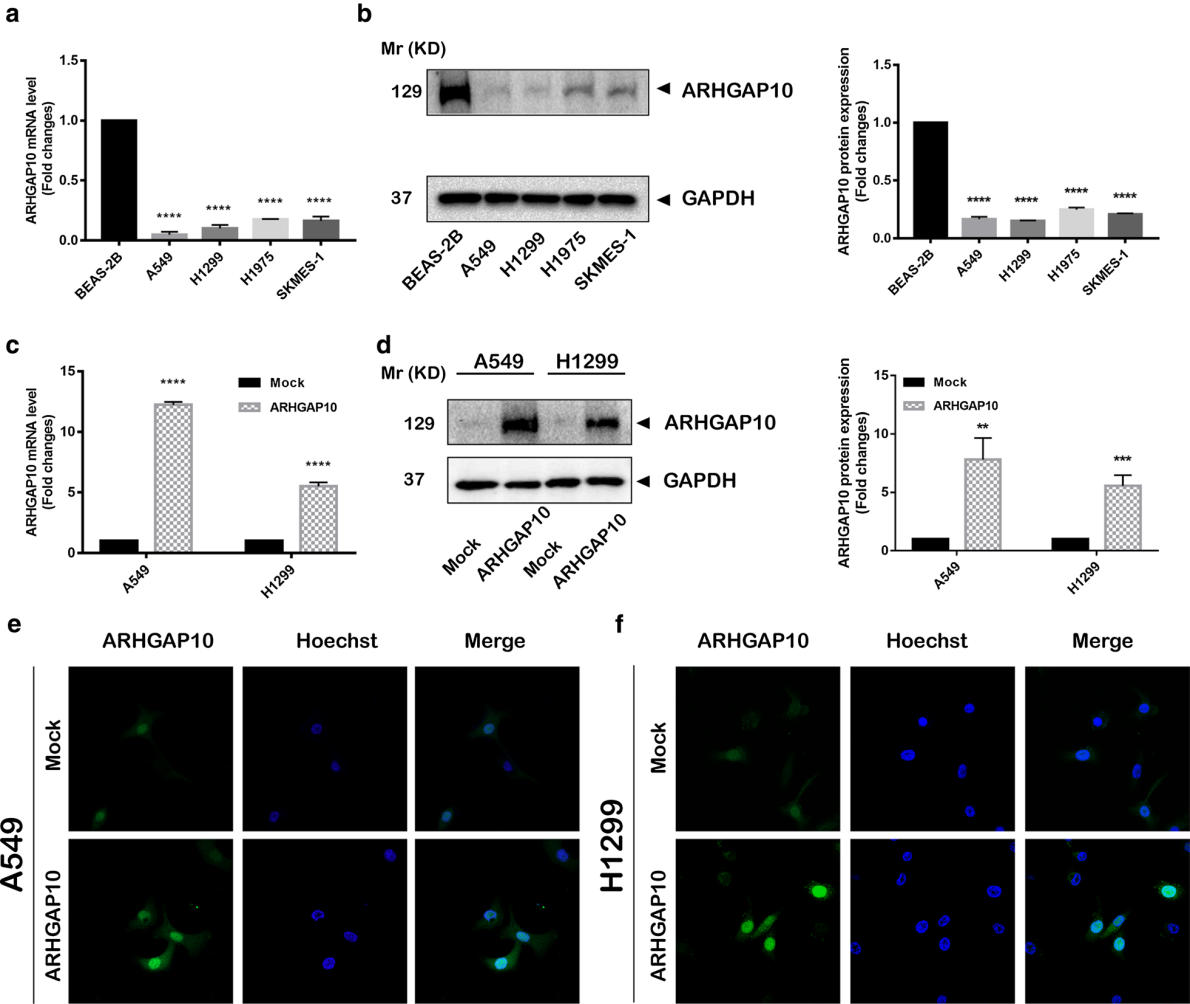
ARHGAP10 expression in NSCLC cells. **a, b** ARHGAP10 expression in NSCLC cell lines was evaluated by RT-qPCR and Western blot. **c**, **d** A549 and NCI-H1299 cells with ARHGAP10 or empty vector stable transfection were identified by RT-qPCR and Western blot. **e**, **f** Immunofluorescence staining was performed to detect the expression and subcellular localization of ARHGAP10 in A549 and NCI-H1299 cells and representative images were taken via laser scanning confocal microscope. (*p < 0.05; **p < 0.01; ***p < 0.001; ****p < 0.0001)

**Fig. 3:**
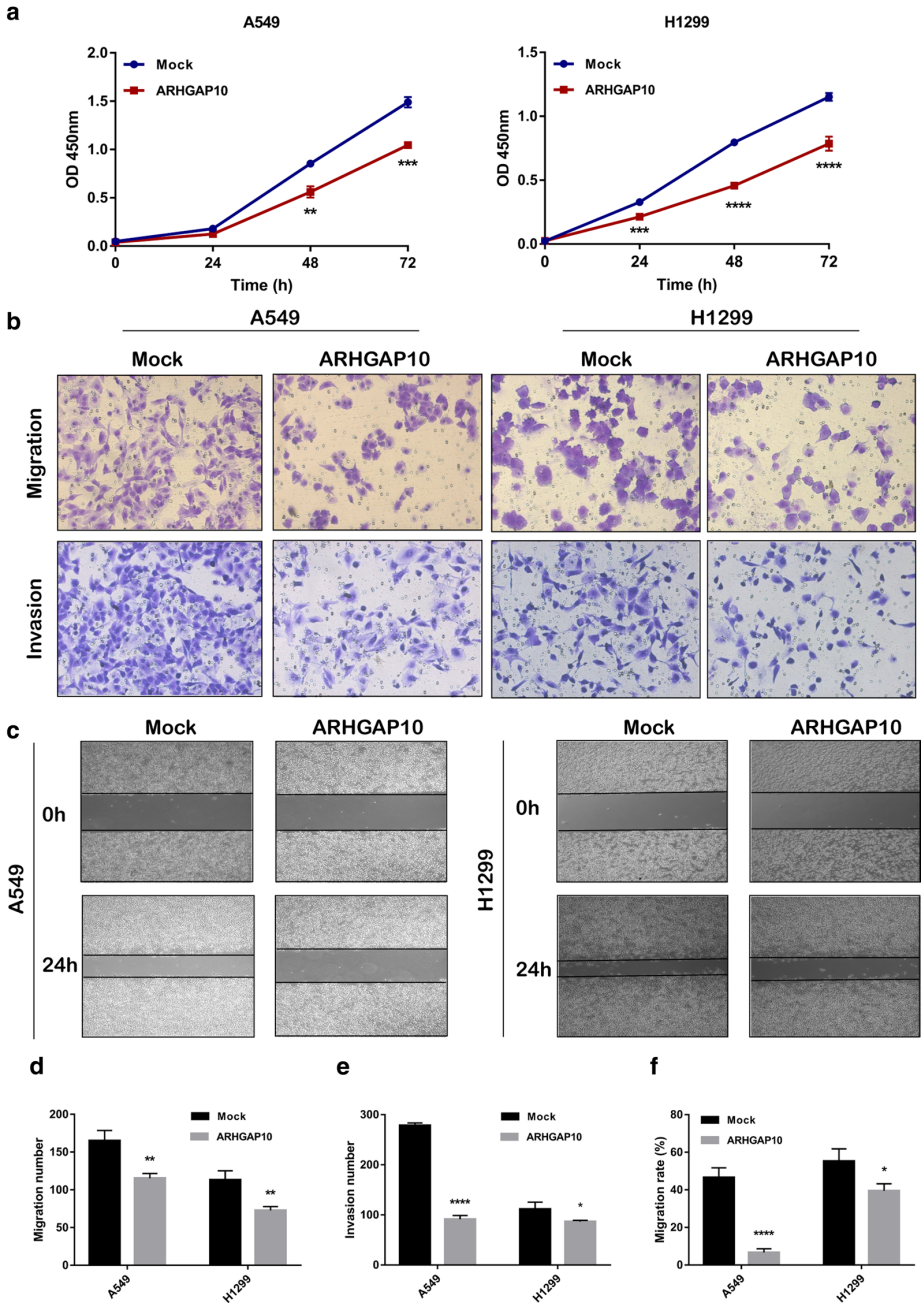
Effect of ARHGAP10 expression on the proliferation, migration and invasion of NSCLC cells. **a** The viability of A549 and NCI-H1299 cells were measured by CCK-8 assays and presented as OD450nm absorbance. **b** The migratory and invasive potentials of A549 and NCI-H1299 cells were determined by Transwell assays. **c** Scratch assays were performed in A549 and NCI-H1299 cells. **d**, **e** Representative images of Transwell migration and invasion assays were shown and the images were photographed at 24 h. **f** Scratch assays were quantified with migration rate and the corresponding images were taken at 0 h and 24 h. (*p < 0.05; **p < 0.01; ***p < 0.001; ****p < 0.0001)

To investigate the effect of ARHGAP10 on NSCLC progression, we detected the ARHGAP10 expression in multiple NSCLC cell lines and lung epithelial cell line BEAS-2B by RT-qPCR and Western blot (Fig. 2a, b), the expression of ARHGAP10 was significantly down-regulated in lung cancer cell lines, especially in the A549 and NCI-H1299 cell lines. Then we established the stable A549 and NCI-H1299 ARHGAP10 overexpression cell lines by lentiviral transduction. As illustrated in Fig. 2c, d, ARHGAP10 mRNA and protein expressions were significantly increased in cells transfected with ARHGAP10 virus compared with vector virus. To further elucidate subcellular localization and expression of ARHGAP10 in NSCLC cell, we performed immunofluorescence assay in A549 and NCI-H1299 cell lines, confocal micrographs displayed that ARHGAP10 localized to the nuclei and cytoplasm region (Fig. 2e, f). Proliferative ability was evaluated using CCK-8 assays. Indeed, the overexpression of ARHGAP10 reduced the viability of NSCLC cells (Fig. 3a). The potentials of migration and invasion were also decreased by overexpression of ARHGAP10 in both A549 and NCI-H1299 cells. These effects were multi-validated by Transwell migration and invasion assays, and scratch assays (Fig. 3b–f).

### ARHGAP10 overexpression cells display altered morphology and actin cytoskeleton appearance and inhibited the process of EMT

**Fig. 4:**
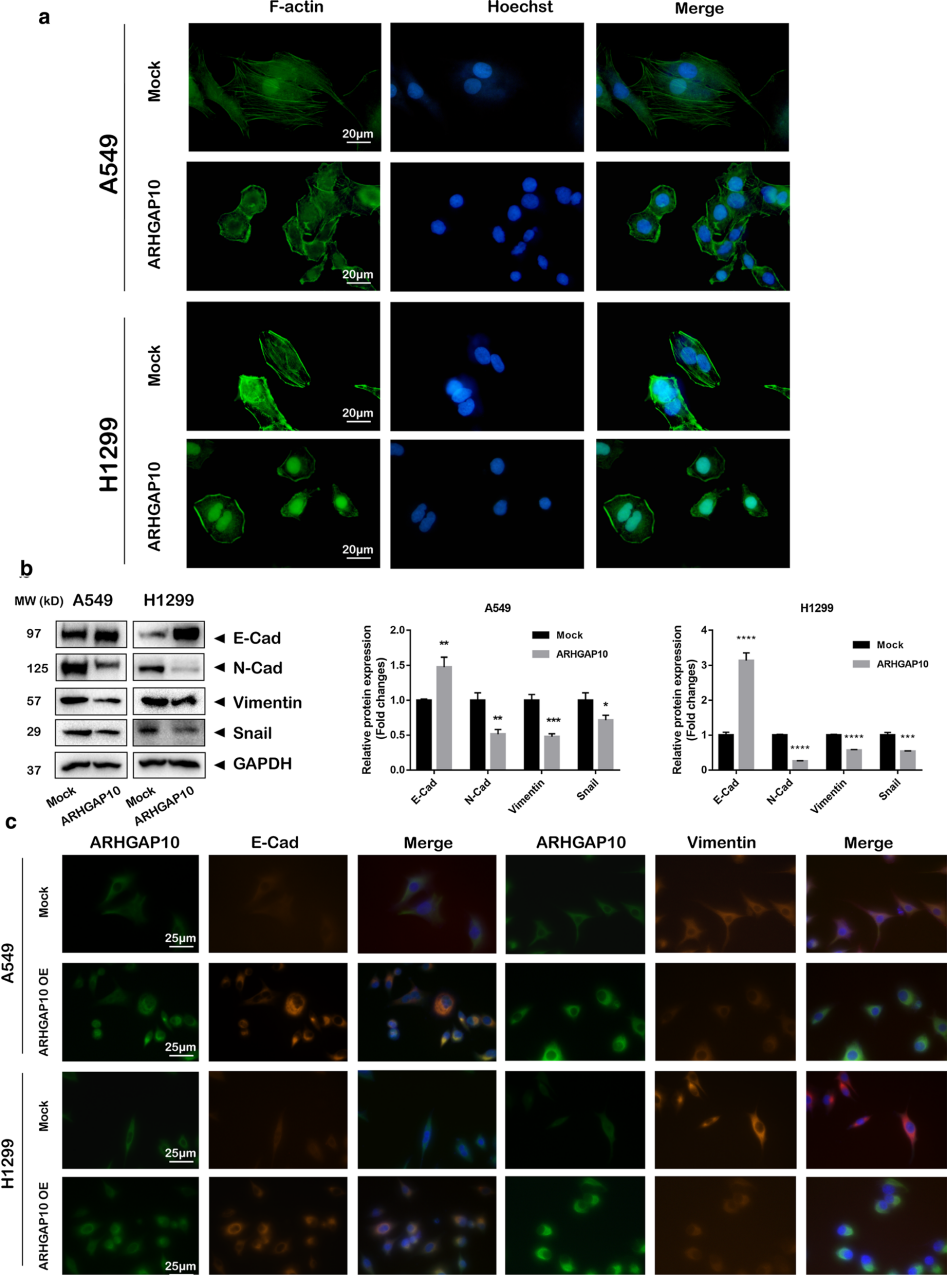
Overexpression of ARHGAP10 altered morphology and inhibit EMT in NSCLC cells. **a** Confocal imaging of actin cytoskeleton stained with phalloidin-FITC presented cellular morphological and cytoskeleton reorganization in A549 and NCI-H1299 cells. Results indicated that actin cytoskeleton changes from disarranged actin to a bundled pattern. **b**, **c** EMT-related proteins E-cadherin, N-cadherin, vimentin and snail were detected by Western blot and immunofluorescence. (*p < 0.05; **p < 0.01; ***p < 0.001; ****p < 0.0001)

Phalloidin was characterized with the property of high affinity with filamentous actin (F-actin) and fluorophore-conjugated phalloidin is an essential tool for cytoskeleton staining in cellular EMT exploration [14, 15]. In present study, phalloidin-FITC stained actin were measured in NSCLC cells. The overexpression of ARHGAP10 triggered morphological changes, and actin cytoskeleton changes from disarranged actin to a bundled pattern displaying non-aggregation of actin in the perinuclear region and the dissociation of membrane protrusions (Fig. 4a). The above phenomena suggested that ARHGAP10 overexpression inhibited epithelial to mesenchymal transition potential. To further investigate the biological function of ARHGAP10 in the progression of EMT, we measured EMT biomarkers by Western blot and immunofluorescence staining, the results illustrated the expression of epithelial marker E-cadherin was obviously increased and mesenchymal markers N-cadherin, snail and vimentin was significantly decreased in ARHGAP10 overexpression cells compared with control groups (Fig. 4b, c).

### ARHGAP10 suppresses EMT in NSCLC cells by PI3K/Akt/GSK3β signaling

**Fig. 5:**
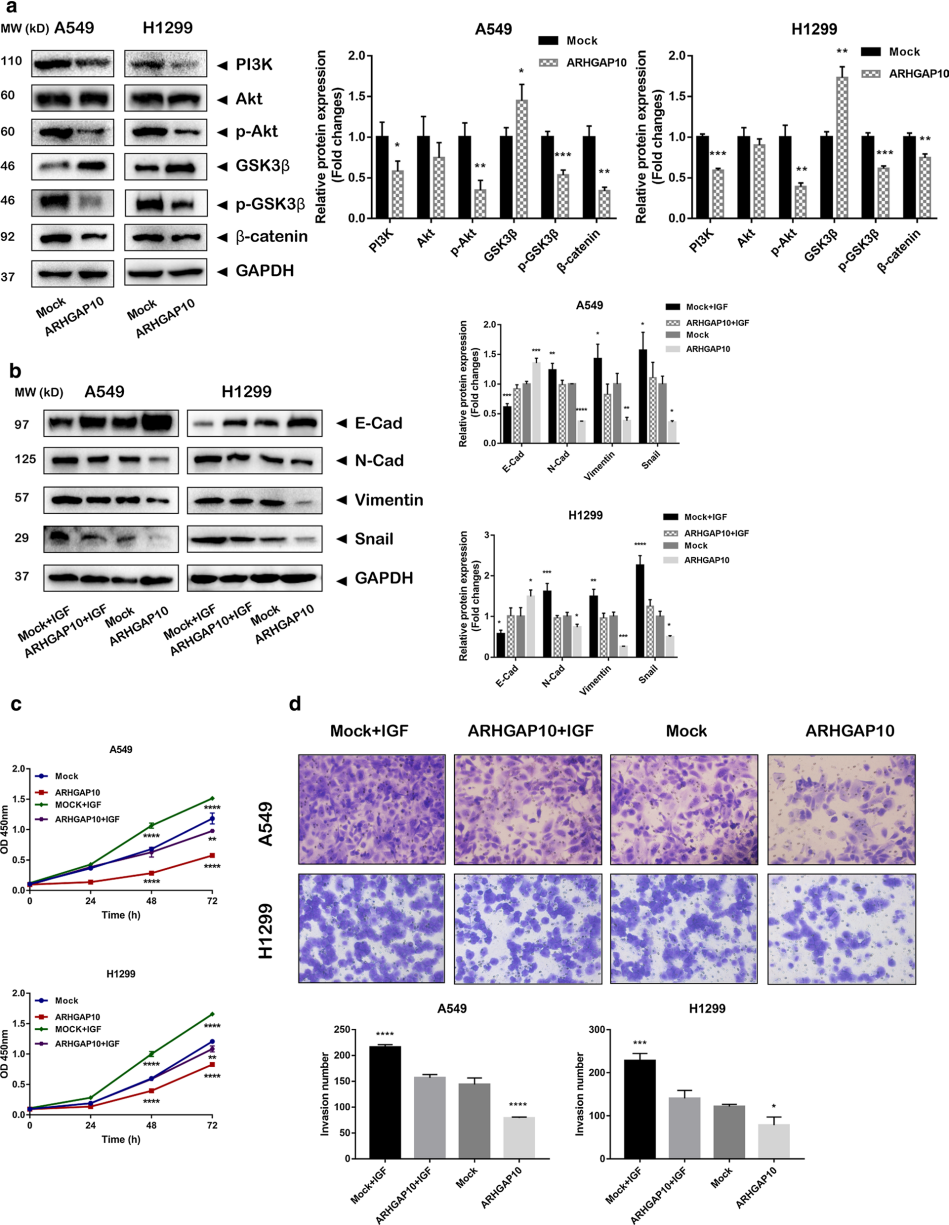
ARHGAP10 suppressed EMT in NSCLC cells by PI3K/Akt/GSK3β signaling. **a** Western blot detected the essential components of PI3K/Akt/GSK3β signaling pathway in A549 and NCI-H1299 cells. **b** Western blot was performed to explore EMT biomarkers expression in A549 and NCI-H1299 cells infected with ARHGAP10 virus or empty vector virus and treated with IGF-1 (100 ng/mL) for 24 h. **c, d** IGF-1 reversed the biological effects of ARHGAP10 on cell proliferation and invasion capabilities in A549 and NCI-H1299 cells. (*p < 0.05; **p < 0.01; ***p < 0.001; ****p < 0.0001)

Kyoto Encyclopedia of Genes and Genomes (KEGG) was performed to excavate signaling pathways that associated with ARHGAP10 expression in the progression of EMT. The results revealed that ARHGAP10 may participate in metastasis and cell cycle via PI3K/Akt signaling pathway. Hence, we further investigated whether PI3K/Akt signaling pathway was involved in the regulation of EMT in ARHGAP10 overexpression cells. The results demonstrated that PI3K, p-Akt (Ser473), p-GSK3β (Ser9) and β-catenin were decreased in ARHGAP10 overexpression cells (Fig. 5a), which revealed that the regulatory effects of ARHGAP10 can be partially attributed to the PI3K/Akt/GSK3β signaling pathways. Moreover, to clarify whether the PI3K/Akt signaling pathway directly mediates the effects of ARHGAP10 on cell metastasis, A549 and NCI-H1299 cells treated with or without ARHGAP10 were exposed to insulin-like growth factors-1 (IGF-1) (100 ng/mL), an activator of PI3K/Akt pathway in tumor progression [16, 17], and evaluated the expression level of EMT biomarkers by Western blot analysis. The results indicated that IGF-1 (100 ng/mL) could reverse the suppression of EMT via ARHGAP10 treatment (Fig. 5b). Moreover, IGF-1 treatment obviously rescued the inhibition of cell proliferation and migration caused by ARHGAP10 overexpression in NSCLC cells (Fig. 5c, d).

## Discussion

Uncontrolled tumor dissemination after oncogenic activation entails profound changes in the gene expression of proteins involved in the cell adhesion process [18]. In the current study, we explored the biological function of ARHGAP10 in NSCLC progression. Our results demonstrated that ARHGAP10 was a remarkable down-expressed gene in NSCLC and predicted poor clinical prognosis. Overexpression of ARHGAP10 contributed to inhibition of EMT, as evidenced by the morphological alternation, upregulation of E-cadherin and downregulation of N-cadherin, snail and vimentin. Emerging evidence has indicated that ARHGAP10 inhibited proliferation and migration of lung cancer [19], but the biological process and potential mechanism of ARHGAP10 in NSCLC epithelial–mesenchymal transition has not been previously verified.

The EMT is a physiological process of cytoskeletal rearrangements and synthesis of extracellular matrix [20], which was characterized as the absence of epithelial cell junctions and polarity, and acquisition of invasive mesenchymal phenotype [21]. Previous studies have reported the involvement of GTPase activation protein in tumorigenesis and metastasis of cancer cells [22–24]. Tian T et al. demonstrated that ARHGAP5, a member of the RhoGAP family, could promote the progression of EMT in colorectal cancer (CRC) cells via negatively regulating RhoA activity, which may serve as a hypothetical theoretical basis of RhoGAP subsequent exploration [25]. In addition, Dai B et al. reported that the protein expression of epithelial biomarker E-cadherin was increased, while expression of the mesenchymal markers N-cadherin and snail were decreased after ARHGAP11A knock-down in hepatocellular carcinoma cells, indicating the role of RhoGAP in regulation of cell conjunction and metastatic process [26].

Since ARHGAP10 was described as an important regulator of actin cytoskeleton dynamics in RhoGAP with well-characterized GAP domains, we supposed ARHGAP10 may be involved in proliferation and metastasis events in NSCLC cells. Previous studies have demonstrated the biological function of ARHGAP10 in malignant tumors. ARHGAP10 has been reported to be downregulated in prostate cancer and may serve as a candidate tumor suppressor [27, 28]. Bigarella et al. reported ARHGAP10 may interacts with the focal adhesion kinase (FAK), a master regulator of integrin signaling known to regulate RhoGAP [29, 30], in glioblastoma cell lines [30]. Luo et al. found that ARHGAP10 was extremely downregulated in ovarian cancer tissues and cell lines, ARHGAP10 was attributed as an essential molecule in controlling many aspects of cell physiology, including cell adhesion, migration and invasion [31]. Additionally, ARHGAP10 overexpression could promote formation of microtubule cytoskeleton [8]. Although the precise mechanism of ARHGAP10 involvement in pulmonary tumor metastasis is unclear, our study showed that ARHGAP10 altered morphology and actin cytoskeleton appearance, suggesting ARHGAP10 could regulate EMT progression in NSCLC.

**Fig. 6:**
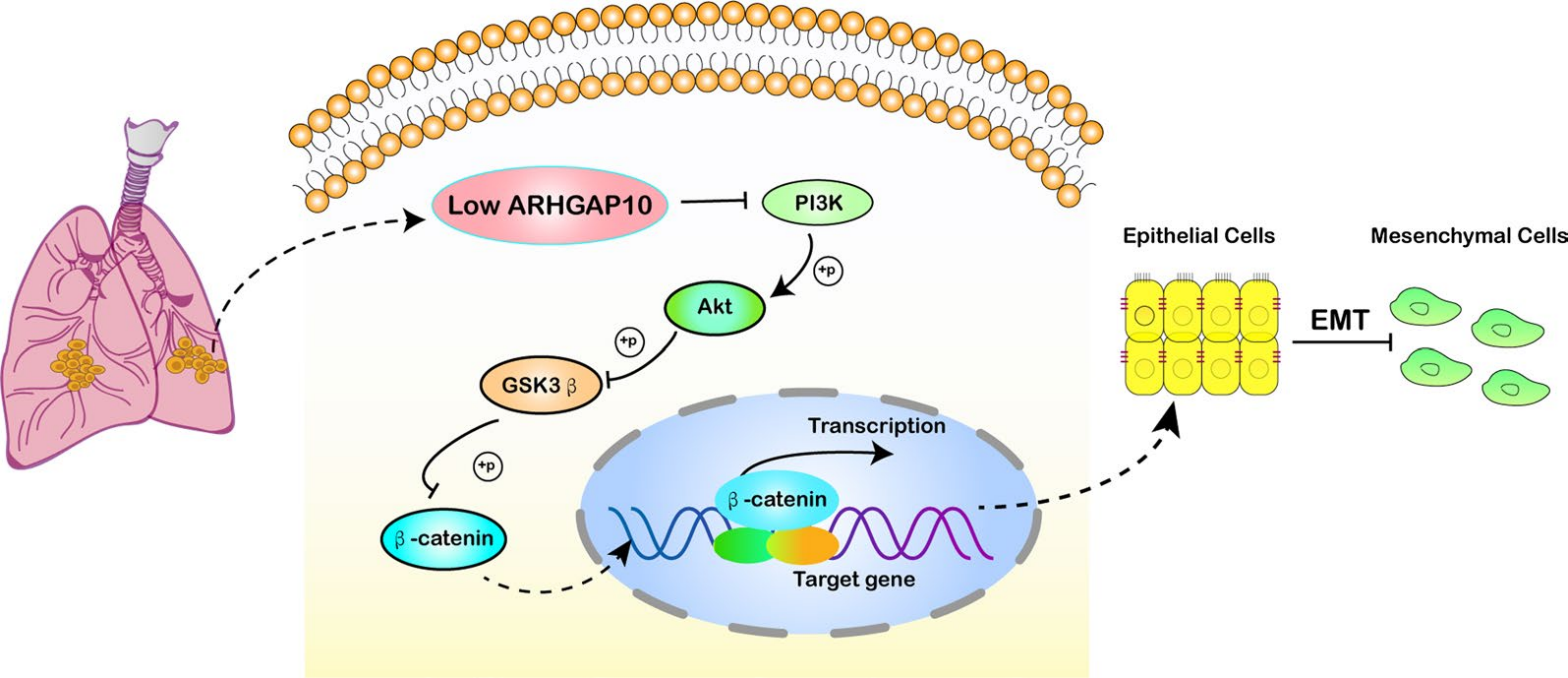
A diagrammatic sketch for the regulatory mechanism of ARHGAP10 in modulating EMT process in NSCLC. Overexpression of ARHGAP10 inhibits PI3K/Akt/GSK3β pathway, resulting in morphological alternation and downregulated EMT biomarkers in NSCLC cells

The aberrant activation of PI3K/Akt pathway can modulate many cellular processes including autophagy, epithelial–mesenchymal transition, apoptosis and metastasis [32]. In this study, we identified a link between the PI3K/Akt pathway and ARHGAP10, showing that ARHGAP10 inhibited NSCLC invasion by modulating the PI3K/Akt/GSK3β pathway. Emerging evidence has indicated that ARHGAP10 suppressed CRC proliferation and metastasis by inhibiting the activity of Rho/Akt signaling pathway [33], consistent with our results that ARHGAP10 down-regulated PI3K/Akt activity in NSCLC cells. The corresponding results indicated that overexpression of ARHGAP10 might inhibit the activation of PI3K. The potential mechanism ARHGAP10 regulated PI3K activity may be attributed to RhoA involvement. The GTPase RhoA is a major regulator of actin reorganization during the formation of stress fibers and can be transformed into inactive pattern by ARHGAP10, a member of RhoGAP [34]. It has been reported that RhoA activated assembly of contractile actomyosin filaments and FAK complex [35]. FAK may activate the PI3K/Akt signaling pathway and induce cellular mobility, migration and invasion [36]. Previous studies have confirmed that PI3K may induce the phosphorylation of the serine residues 308 and 473 of Akt [37, 38]. Phosphorylated Akt (p-Akt) can phosphorylate GSK3β and inhibit its kinase activity, leading to the inactivation of GSK3β [39]. Decreased GSK3β alleviates restraint on β-catenin [40], contributing to the accumulated β-catenin entry into the nucleus to activate gene transcription and interacts with TCF/LEF and ultimately triggers the transcription of target genes including snail [41, 42]. As a direct downstream gene of snail, the expression of epithelial biomarker E-cadherin was significantly downregulated (Fig. 6). In present research, ARHGAP10 can decrease the expression levels of constituents of PI3K/Akt/GSK3β pathway and subsequently downregulated EMT biomarkers expression in lung cancer.

The aim of the present study was to evaluate the biological effect and potential mechanism of ARHGAP10 in vitro experiments as an initial exploration. However, we need to acknowledge that there were some limitations in our study. Firstly, there was a limited quantity of clinical samples and survival information of clinical patients was not collected to predict prognosis, which is partly due to the access of specimen and data. Secondly, the direct combined molecule of ARHGAP10 participating in EMT was not detected and the profound mechanism of ARHGAP10 regulating the process of cancer metastasis was not revealed. In addition, experimental animal models of Xenograft tumor formation were not conducted, which need to be further explored.

## Conclusion

In summary, our current study documented that ARHGAP10 was remarkably downregulated in NSCLC cells and predicted poor clinical prognosis. Overexpression of ARHGAP10 could inhibit the progression of EMT by PI3K/Akt/GSK3β pathway. Our results indicated agonist of ARHGAP10 may be an optional remedy for NSCLC patients than traditional opioids.

## Data Availability

All data generated or analyzed in present study are included in this published article, and its additional information files.

## Abbreviations

ARHGAP: Rho GTPase activating protein
BSA: Bovine serum albumin
CCK-8: Cell Counting Kit-8
CRC: Colorectal cancer
EMT: Epithelial–mesenchymal transition
F-actin: Filamentous actin
FBS: Fetal bovine serum
IF: Immunofluorescence
IGF-1: Insulin-like growth factors-1
IHC: Immunohistochemistry
KEGG: Kyoto Encyclopedia of Genes and Genomes
MOI: Multiple of infection
NSCLC: Non-small cell lung cancer
OE: Overexpression
PBS: Phosphate buffered saline
PH: Pleckstrin homology
RT‑qPCR: Reverse transcription‑quantitative PCR
RhoGAP: Rho GTPase activating protein
